# Mitochondrial respiration in human peripheral blood mononuclear cells: methodology and influence of permeabilization and storage

**DOI:** 10.3389/fmolb.2026.1756657

**Published:** 2026-03-13

**Authors:** Shusuke Sekine, Imen Chamkha, Evelina Elmér, Eleonor Åsander Frostner, Emil Westerlund, Tianshi Liu, Johannes Ehinger, Fredrik Sjövall, Hiroyuki Uchino, Eskil Elmér

**Affiliations:** 1 Department of Clinical Sciences Lund, Mitochondrial Medicine, Lund University, Lund, Sweden; 2 Department of Anesthesiology, Tokyo Medical University, Tokyo, Japan; 3 Division of Transfusion Medicine, Department of Laboratory Medicine, Lund University, Lund, Sweden; 4 Department of Clinical Immunology and Transfusion Medicine, Skåne University Hospital, Lund, Sweden; 5 Emergency Department, Kungälv Hospital, Kungälv, Sweden; 6 Department of Clinical Sciences Lund, Otorhinolaryngology, Head and Neck Surgery, Lund University, Lund, Sweden; 7 Department of Intensive- and perioperative Care, Skåne University Hospital, Malmö, Sweden; 8 Clinical Neurophysiology, Medical Imaging and Physiology, Skåne University Hospital, Lund, Sweden

**Keywords:** digitonin, electron transfer system, lymphocytes, Oroboros, peripheral blood mononuclear cells, permeabilization, respirometry, substrate–uncoupler–inhibitor titration protocol

## Abstract

Human peripheral blood mononuclear cells (PBMCs) can be easily sampled from healthy individuals and patients. Density gradient isolation from human blood or leukocyte concentrates yields a mononuclear cell population of mainly lymphocytes, monocytes, and natural killer (NK) cells. PBMCs are vital circulating cells of the immune system and rely on oxidative phosphorylation (OXPHOS) for their energy production. OXPHOS capacity can be assessed using oxygraphy in intact and permeabilized PBMCs and has been used to investigate disorders of the immune system, but also, similarly to platelets, employed as a bioenergetic biomarker, that is, a “liquid biopsy” of disease conditions unrelated to immune dysregulation. Here, we present some key aspects of mitochondrial respiration in PBMCs isolated from leukocyte concentrates and whole blood using the Oroboros O2k oxygraph. We assessed the limits of sample amount and the impact of storage time and temperature and explored critical aspects of digitonin permeabilization. Furthermore, we provide respiratory rates and internal ratios from healthy controls using simple and comprehensive protocols for intact and permeabilized PBMCs, respectively. We conclude that detailed information on OXPHOS capacity in PBMCs can be reproducibly assessed *ex vivo*, but that great care must be taken during permeabilization to achieve correct measures of respiratory rates.

## Introduction

Peripheral blood mononuclear cells (PBMCs) represent a heterogeneous, readily accessible population of circulating immune cells. They can be acquired through venipuncture, which facilitates repeated sampling and enhances feasibility for large-scale studies. Density gradient isolation from human blood or leukocyte suspensions yields a mononuclear cell population typically comprising a mixture of mainly lymphocytes (80%–90%), monocytes (10%–20%), and natural killer (NK) cells (Timon-Gomez, 2025; Sumbalova, 2020).

PBMCs rely on ATP produced via oxidative phosphorylation (OXPHOS) for activation, proliferation, and effector responses (Chacko et al., 2013). High-resolution respirometry (HRR), or oxygraphy, enables precise assessment of mitochondrial respiratory rates, coupling efficiency, and electron transfer system (ETS) integrity (Timon-Gomez, 2025; Doerrier et al., 2018; Gnaiger, 2020b; Gnaiger, 2020a). Moreover, measurements performed in intact cells preserve cellular architecture, signaling context, and metabolic compartmentalization, thereby more closely approximating *in vivo* physiology than assays relying on isolated mitochondrial preparations. Furthermore, respiration of intact cells can be assessed in physiologically relevant surrounding media. For circulating blood cells, oxygraphy is optimal as cells are submerged and “circulated” in an excess of extracellular buffer of choice, such as the person’s own plasma for intact cells, or a “cytoplasmic” buffer with composition optimized for permeabilized cells, such as the Oroboros respiration medium MiR05 (Baglivo, 2024). Gentle permeabilization of the cell membrane makes it possible for non-permeable substrates and inhibitors to access the mitochondria directly, and pathways and even individual complexes of the ETS can be investigated without the need for forceful cell disruption and mitochondrial purification (Timon-Gomez, 2025; Gnaiger, 2020b; Gnaiger, 2020a). Selected publications assessing mitochondrial respiration of PBMCs using oxygraphy are listed on Bioblast MitoPedia, an open-access knowledge database maintained by Oroboros Instruments (https://www.bioblast.at/index.php/PBMC).

The PBMC population has frequently been used to assess bioenergetic phenotypes of the immune system (immunometabolism), but significant challenges remain (Macleod, 2025). Variability in PBMC isolation procedures and buffers used, phenotypic composition of samples including contaminating platelets (Sumbalova, 2020; Rausser et al., 2021), as well as differences in respirometric protocols employed (e.g., concentrations of substrates and inhibitors used and the order of additions) are factors that can influence results and comparability between studies. For example, clear differences have been demonstrated in mitochondrial activity across immune cell subtypes of the PBMC population (Rausser et al., 2021), and the choice of respiration medium can affect the HRR of intact human PBMCs by influencing cell count and aggregation propensity (Bager Christensen et al., 2024).

It is becoming clear that respirometry of PBMCs (or platelets) should not be used as a universal biomarker for less accessible tissues, or as a signature for systemic mitochondrial function in healthy controls (Rausser et al., 2021; Westerlund et al., 2024; Devine et al., 2025). However, for systemic mitochondrial disease with a high mutation load also in blood cells, drug intoxications, poisonings, or other conditions directly affecting mitochondrial function of the whole organism, blood cell respirometry has potential as a minimally invasive diagnostic tool. The method is suitable for repeated sampling, could correlate with disease severity, and could be used to monitor therapeutic intervention (Westerlund et al., 2024; Sjovall et al., 2014; Sjovall et al., 2010; Sjovall et al., 2013a; Westerlund et al., 2018; Bungatavula et al., 2025; Jang et al., 2018; Weiss et al., 2022; Lindeman et al., 2026).

Here, to highlight some critical aspects of the analysis of respiratory capacity in PBMCs, we present our long-term experience using the Oroboros O2k oxygraph in PBMCs isolated from leukocyte concentrates and whole blood. We assessed the limits of sample amount, the impact of storage time and temperature, and explored critical aspects of digitonin permeabilization. Furthermore, we provide updated control values for respiratory rates and selected ratios from our specific protocols for intact and permeabilized PBMCs, respectively.

## Materials and methods

### Human sample collection

The study was approved by the Regional Ethical Review Board of Lund, Sweden (approval numbers 113/2008 and 644/2009) and the Swedish Ethical Review Authority (2019-04947 and 2023–04796-02). Written informed consent was obtained from all participants prior to blood sample collection. Leukocyte suspensions were acquired from the Clinical Immunology and Transfusion Medicine Department at Skåne University Hospital under permit numbers 2022:23 and 2024:17.

Unless otherwise indicated, all chemicals were purchased from Sigma-Aldrich (St. Louis, MO, United States).

### Blood collection and leukocyte preparation

Leukocyte concentrates were obtained from 400 mL of whole blood processed using the Reveos 3C System® (Terumo Blood and Cell Technology, United States) as part of the routine blood donation procedure at the Department of Clinical Immunology and Transfusion Medicine, Skåne University Hospital, Lund, Sweden. The Reveos system isolates blood components through automated, high-speed centrifugation of whole blood, separating it by density into plasma, platelets, red blood cells, and a leukocyte concentrate. The concentrate consists mainly of leukocytes dissolved in plasma with citrate phosphate dextrose (CPD-anticoagulant) and the Reveos AS-5 cell preservative solution. For other participants, 36 mL of venous blood was collected into K_2_EDTA tubes (BD Vacutainer®, Becton Dickinson and Company, Plymouth, United Kingdom). PBMCs isolated from leukocyte suspensions obtained using the Reveos 3C System® within 8 h after blood donation displayed similar respiratory capacities (rates and ratios) for intact and permeabilized cells as PBMCs freshly isolated from whole blood (Supplementary Figure S1, S2).

### Isolation of peripheral blood mononuclear cells (PBMCs)

PBMCs were isolated from leukocyte concentrates or whole blood within 8 h, 24 h, or 48 h of collection. For experiments addressing the effects of delayed isolation following collection, samples were stored on a tilting board at room temperature or +4 °C. Samples were diluted 1:1 with 0.9% NaCl (saline) and carefully layered onto Ficoll-Paque (Lymphoprep®, STEMCELL Technologies, Canada). After centrifugation at 800 *g* for 30 min at room temperature (RT), the PBMC layer at the Ficoll interface was collected and transferred to a 50 mL tube. It was then diluted 10-fold with saline and centrifuged once at 250 *g* for 10 min at RT. The resulting pellet was resuspended in ∼1 mL of saline and mixed with plasma at a 2:1 saline-to-plasma ratio.

To determine cell counts, 4 µL of the PBMC suspension was diluted with 36 µL of saline and introduced into a capillary tube. Cell counts were obtained using a Swelab Alfa Plus Standard hematology analyzer (Boule Diagnostics AB, Spånga, Sweden).

### High-resolution respirometry

Mitochondrial respiration was analyzed at 37 °C using high-resolution respirometers (Oxygraph-2k, Oroboros Instruments, Innsbruck, Austria), as described previously (Doerrier et al., 2018; Gnaiger, 2020b; Gnaiger, 2020a; Sjovall et al., 2010). Respirometry experiments were performed in 2 mL glass chambers equipped with magnetic stirrers (750 rpm). Oxygen concentration was maintained above 50 μM, and chambers were briefly opened for reoxygenation when required. Instrumental background oxygen flux was determined in separate experiments and automatically corrected for according to the manufacturer’s protocol.

Daily air calibration was performed using Millipore water equilibrated with air until a stable signal was achieved. Oxygen concentrations were automatically calculated from barometric pressure and oxygen solubility factors (1.0 for water; 0.92 for MiR05) (Doerrier et al., 2018).

All respirometry assays were conducted in glucose-free MiR05 medium (Oroboros Instruments, Innsbruck, Austria) containing 110 mM D-sucrose, 60 mM K-lactobionate, 20 mM HEPES, 20 mM taurine, 10 mM KH_2_PO_4_, 3 mM MgCl_2_, 0.5 mM EGTA, and 1 g/L fatty acid-free BSA (pH 7.1). PBMC concentration in the oxygraph chamber ranged from 1 × 10^6^ cells/mL to 10 × 10^6^ cells/mL.

### Measurement of oxygen consumption in intact PBMCs

Oxygen consumption in intact PBMCs was analyzed in the MiR05 respiration medium using endogenous substrates. ROUTINE respiration, representing basal oxidative phosphorylation (OXPHOS) under physiological substrate supply, was recorded over a 10–15 min stabilization period following chamber closure. Oligomycin (1 μg/mL) was then added to inhibit ATP synthase and determine LEAK respiration. Maximal capacity of the electron transfer system (ETS) was assessed by stepwise titration of the protonophore carbonyl cyanide 4-(trifluoromethoxy)phenylhydrazone (FCCP) (0.5 µM/step) until oxygen flux reached a plateau. Rotenone (2 μM; complex I inhibitor) and antimycin A (1 μg/mL; complex III inhibitor) were subsequently added to determine residual oxygen consumption (Rox), which was subtracted from all respiratory rates.

### Optimization of digitonin permeabilization

The optimal digitonin concentration for PBMC permeabilization was determined as described by Doerrier et al. (2018). PBMCs (10 × 10^6^ cells/mL) were pre-incubated with rotenone (2 µM) and succinate (10 mM) in the presence (or absence) of ADP (1 mM). Digitonin (5 μg/μL stock) was titrated stepwise until maximal oxygen flux was achieved and continued until clear inhibition of respiration was visible (the latter only in experiments with both ADP and succinate present).

### Measurement of oxygen consumption in digitonin-permeabilized PBMCs

The respiratory capacities of permeabilized PBMCs were evaluated using a substrate–uncoupler–inhibitor titration (SUIT) protocol (Timon-Gomez, 2025; Gnaiger, 2009). Following digitonin permeabilization, malate (5 mM) and pyruvate (5 mM) were added, followed by ADP (1 mM) and glutamate (5 mM) to determine the OXPHOS capacity of the NADH-linked pathway (N_*P*_). Succinate (10 mM) was then added to assess the OXPHOS capacity of the convergent NADH- and succinate-linked pathway (NS_*P*_). Oligomycin (1 μg/mL) was added to induce LEAK respiration of the convergent NADH- and succinate-linked pathway (NS_*L*_), and FCCP was titrated stepwise (0.5 µM/step) to determine the electron transfer (ET) capacity of the convergent NADH- and succinate-linked pathway (NS_*E*_). Rotenone (2 µM) was then added to inhibit complex I and determine the ET capacity of the isolated succinate-linked pathway (S_*E*_), followed by antimycin A (1 μg/mL) to inhibit complex III and determine Rox.

### Flow cytometry

PBMCs were isolated (see above) from leukocyte suspensions within 8 h after a blood donation and isolated again from the same suspensions after 48 h of storage. Samples were analyzed using flow cytometry at the Department of Clinical Immunology and Transfusion Medicine, Skåne University Hospital, Lund. In brief, an antibody cocktail of the following fluorochrome-conjugated monoclonal antibodies (Beckman Coulter, Brea, CA, United States) was added to the suspension of isolated PBMCs: CD45 ECD (J33), CD3 APC–Alexa Fluor 750 (UCHT1), CD4 APC (13B.8.2), CD8 APC–Alexa Fluor 700 (B9.11), CD19 PC5.5 (J3-119), CD16 PE (3G8), and CD56 PE (N901). Acquisition was performed on a Navios 8 flow cytometer (Beckman Coulter), and data were analyzed using Kaluza Analysis Software version 2.1 (Beckman Coulter). At least 5,000 lymphocytes were acquired. Initially, CD45^+^ cells were divided into monocytes, lymphocytes, and granulocytes based on forward and side scatter properties. Analysis using this antibody cocktail allowed identification of NK cells (CD3^−^CD16^+^CD56^+^ lymphocytes), B cells (CD19^+^ lymphocytes), and T cells (CD3^+^ lymphocytes) and their subsets: helper T cells (CD4^+^) and cytotoxic T cells (CD8^+^). The percentages of lymphocytes and non-lymphocytes of the total number of CD45^+^ cells were calculated. Due to technical issues with the flow cytometer, two samples were analyzed only at the first time point.

### Data analysis

Statistical analyses were performed using GraphPad Prism version 10.02 (GraphPad Software, La Jolla, CA, United States). Data are presented as median (IQ range) or as individual values. All oxygen fluxes were corrected for Rox (Doerrier et al., 2018). Comparisons of paired data between two groups were conducted using the Wilcoxon test, while multiple-group comparisons were analyzed using Friedman’s test, a non-parametric ANOVA, with post-hoc Dunn’s multiple comparisons test. A p-value <0.05 was considered statistically significant.

## Results

### Mitochondrial respiration in intact PBMCs

**FIGURE 1:**
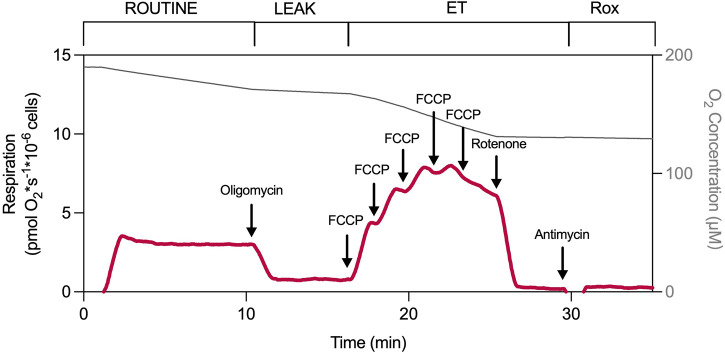
Experimental protocol of intact PBMCs. Mitochondrial respiration was measured in intact PBMCs by high-resolution respirometry using a coupling-control protocol with consecutive inhibitor additions and uncoupler titration (Timon-Gomez, 2025; Gnaiger, 2020b; Gnaiger, 2020a; Gnaiger, 2009). After establishing endogenous (ROUTINE) respiration, LEAK respiration was induced by adding the ATP synthase inhibitor oligomycin (1 μg/mL). Electron transfer pathway capacity (ET capacity), reflecting the maximally decoupled respiration, was determined by titration of the protonophore, carbonyl cyanide p-(trifluoromethoxy)phenylhydrazone (FCCP). Addition of rotenone (2 µM) followed by antimycin A (1 μg/mL), complex I and complex III inhibitors, respectively, revealed residual oxygen consumption (Rox). Induced respiratory coupling states, uncoupler, and inhibitors used are indicated in and above the graph.

The mitochondrial electron transfer system (ETS) of intact human PBMCs was evaluated by high-resolution respirometry (Figure 1; Table 1). Mitochondrial respiration in the resting state (ROUTINE respiration) is mainly driven by endogenous substrates reflecting basal ATP demand. When inhibiting the ATP synthase by oligomycin, the phosphorylating system halts, and a basal “LEAK” state is established, primarily reflecting proton leak over the inner mitochondrial membrane but also proton slip, cation cycling, and electron leak (Gnaiger, 2020a). Applying an uncoupler disrupts the electrochemical gradient across the inner mitochondrial membrane, stimulating the ETS to reach its maximal decoupled capacity (ET capacity). The complex I inhibitor, rotenone, and the complex III inhibitor, antimycin A, fully inhibit the ETS and reveal any residual oxygen consumption (Rox) of the cell (Gnaiger, 2020b; Gnaiger, 2020a; Gnaiger, 2009). Selected respiratory parameters of PBMCs freshly isolated from whole blood from an adult control cohort (n = 26) are provided in Table 2.

**TABLE 1:** Mitochondrial respiratory rates at different coupling-control and pathway-control states measured by high-resolution respirometry and the corresponding respiratory ratios used in this study. Adopted from Gnaiger (2020b) and Gnaiger (2020a), where additional rates, ratios, and states are described in detail.

Respiratory parameter	Abbreviation	Explanation
ROUTINE respiration	*R*	The physiological oxygen consumption in intact cells representing the control of cellular substrate uptake and energy turnover
LEAK respiration	*L*	Background respiration (with oligomycin-inhibited ATP synthase), with endogenous substrates (intact cells) or pyruvate, malate, glutamate, and succinate (permeabilized cells). Consists mainly of proton leak over the inner mitochondrial membrane
Electron transfer (ET) capacity	*E*	Maximal decoupled respiration at saturating concentrations of an uncoupler, with endogenous substrates (intact cells) or pyruvate, malate, glutamate, and succinate (permeabilized cells)
Residual oxygen consumption	*Rox*	The oxygen consumption due to oxidative side reactions, measured after inhibiting the mitochondrial electron transfer system (ETS)
Electron transfer (ET) capacity of the succinate-linked pathway	S_*E*_	Maximal decoupled respiration with succinate
Electron transfer (ET) capacity of the convergent NADH- and succinate-linked pathway	NS_*E*_	Maximal decoupled respiration with pyruvate, malate, glutamate, and succinate
LEAK respiration of the convergent NADH- and succinate-linked pathway	NS_*L*_	Background respiration (with oligomycin-inhibited ATP synthase) with pyruvate, malate, glutamate, and succinate. Consists mainly of proton leak over the inner mitochondrial membrane
OXPHOS capacity of the convergent NADH- and succinate-linked pathway	NS_*P*_	Maximal ADP-stimulated respiration with pyruvate, malate, glutamate, and succinate
OXPHOS capacity of the NADH-linked pathway	N_*P*_	Maximal ADP-stimulated respiration with pyruvate, malate, and glutamate
Uncoupling-control ratio (*E*/*L*)	*E*/*L*	A flux control ratio reflecting the mitochondrial coupling efficiency in intact cells
Uncoupling-control ratio (*E*/*R*)	*E*/*R*	A flux control ratio of how far the ET capacity (*E*) is from the endogenous ROUTINE respiration (*R*)
R-L control efficiency	1−*L*/*R*	A coupling-control efficiency reflecting the effectiveness of ROUTINE respiration (*R*) in intact cells
Respiratory acceptor control ratio (RCR)	*P*/*L*	A flux control ratio reflecting the degree of respiratory control exerted by ADP (the “acceptor”)
P-L control efficiency	1−*L*/*P*	A coupling-control efficiency reflecting the fraction of OXPHOS capacity (*P*) that is coupled to net energy turnover (primarily ATP production), relative to the total maximum capacity

**TABLE 2:** Respiratory rates and ratios of intact PBMCs isolated from freshly drawn whole blood from healthy volunteers.

Selected respiratory parameter	Adult participant (age ≥18 years)
ROUTINE (*R*)	4.30 (1.81)
LEAK (*L*)	0.59 (0.30)
ET (*E*)	12.79 (3.07)
*E*/*R*	2.95 (1.25)
*E*/*L*	19.94 (19.32)
1−*L*/*R*	0.87 (0.08)

^a^ Respiration measured in pmol O_2_/s /10^6^ PBMCs, except for ratios that are independent of cell count and mitochondrial content; IQR, interquartile range. For definitions of respiratory parameters, see Table 1. n = 26.

### Evaluation of chamber cell concentration on mitochondrial respiration in intact cells

**FIGURE 2:**
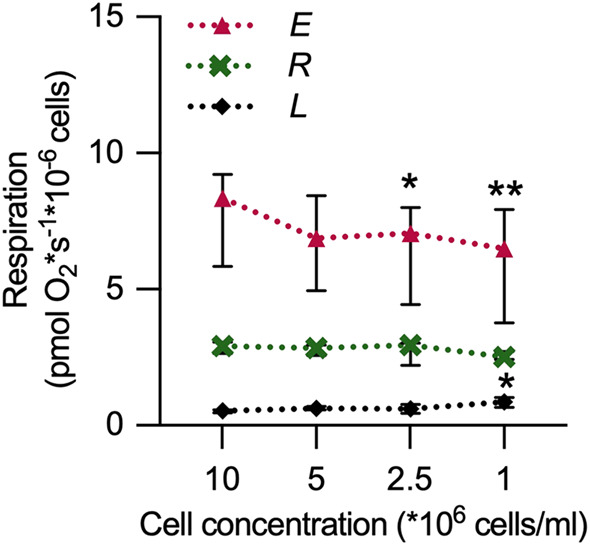
The oxygraph chamber concentration of PBMCs affects the determination of electron transfer pathway capacity (*E*) and LEAK respiration of intact cells. Mitochondrial respiratory rates were measured in intact PBMCs, using high-resolution respirometry, at three different respiratory coupling states induced in a coupling-control protocol at PBMC concentrations ranging from 1 × 10^6^ cells/mL to 10 × 10^6^ cells/mL. At ≤ 2.5 × 10^6^ cells/mL, the ET capacity was significantly lower than at 10 × 10^6^ cells/mL. In addition, LEAK respiration was significantly higher at 1 × 10^6^ PBMCs/mL. For definitions of respiratory rates, see Table 1. *p < 0.05 compared to the 10 × 10^6^ PBMCs/mL group. **p < 0.01 compared to the 10 × 10^6^ PBMCs/mL group. Data were analyzed by the Friedman test followed by Dunn’s multiple comparison test and plotted as median with interquartile range (IQR). n = 6.

The mitochondrial respiration per million cells at decreasing PBMC concentrations in the oxygraph chambers was evaluated. At 1–2.5 × 10^6^ PBMCs/mL, the ET capacity was significantly lower than at 10 × 10^6^ PBMCs/mL. At the same time, LEAK respiration was significantly higher at 1 × 10^6^ PBMCs/mL (Figure 2). Based on this, subsequent experiments were performed at ≥5 × 10^6^ PBMCs/mL.

### The influence of storage on respiratory parameters of intact PBMCs

**FIGURE 3:**
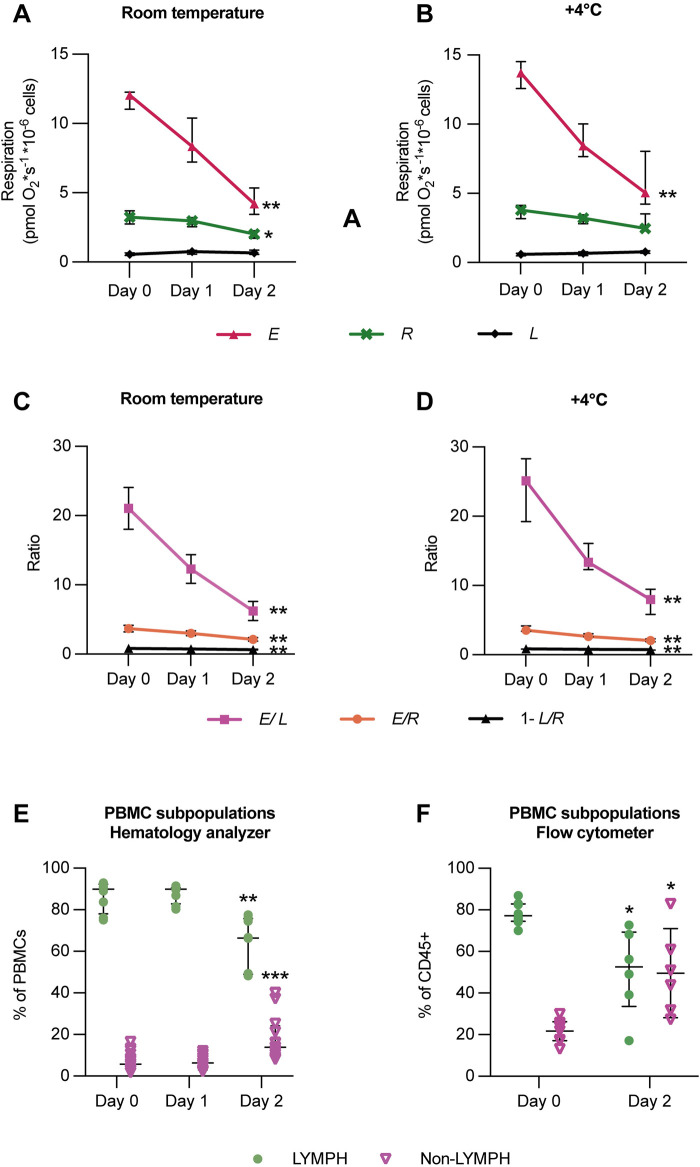
Storage of PBMCs over 2 days decreases mitochondrial respiratory rates in intact cells and changes the resulting phenotype of immune cells following density gradient isolation. Leukocyte suspensions were stored at **(A,C)** room temperature or **(B,D)** +4 °C on a tilting board. PBMCs were isolated and analyzed immediately (Day 0), after 1 day (Day 1), and after 2 days (Day 2). Mitochondrial respiratory rates were measured in intact PBMCs using high-resolution respirometry. Results indicate that delayed isolation following storage (regardless of storage temperature) lowered mitochondrial respiratory rates and selected flux control ratios and efficiencies **(C,D)**. n = 6. Furthermore, storing leukocyte suspensions over 2 days changed the resulting phenotype of immune cells following isolation, when quantified using an automated hematology analyzer **(E)** and using flow cytometry **(F)**. Storage for 48 h resulted in a decreased proportion of lymphocytes and an increased proportion of non-lymphoid cells of the isolated PBMC population, n = 8. Respirometry and cell counting data were analyzed by the Friedman test followed by Dunn’s multiple comparison test, and flow cytometry data were analyzed using the Wilcoxon matched-pairs signed-rank test and plotted as median with interquartile range (IQR). *p < 0.05, **p < 0.01, ***p < 0.001. For definitions of respiratory rates and ratios, see Table 1.

The effect of storage on the mitochondrial respiration of intact PBMCs was primarily evaluated in PBMCs stored on a tilting board as leukocyte suspensions (consisting of mainly leukocytes dissolved in plasma with citrate phosphate dextrose (CPD-anticoagulant)) obtained from 400 mL of whole blood processed using the Reveos 3C System® (Figure 3). At room temperature, the absolute rates of ROUTINE respiration, ET capacity, and the uncoupling-control ratios (*E*/*L)* and (*E*/*R)* all significantly decreased when PBMCs were isolated from the suspensions following 2 days of storage, compared to cells isolated from suspensions < 8 h from blood donation (Figures 3A,C). Similarly, at 4 °C, except for ROUTINE respiration, the ET capacity (*E*) and the uncoupling-control ratios (*E*/*L)* and (*E*/*R)* were significantly lower after 2 days of storage (Figures 3B,D). Furthermore, the R-L control efficiency (1−L/R), that is, the fraction of routine respiration coupled to phosphorylation in intact cells, also decreased with storage (Figures 3C,D). Similar alterations in rates and ratios due to storage were obtained in PBMCs stored and isolated from whole blood resting at room temperature (data not shown).

### The influence of storage on the resulting PBMC phenotype following isolation

The storage of leukocyte suspensions over 2 days altered the resulting phenotype of the PBMC population following isolation. Regardless of the storage temperature applied, the fraction of lymphocytes was decreased, and the fraction of other PBMC subpopulations, primarily monocytes and granulocytes, was increased after 2 days, when quantified using an automated hematology analyzer and using flow cytometry (Figures 3E,F).

### Mitochondrial respiration of permeabilized PBMCs

**FIGURE 4:**
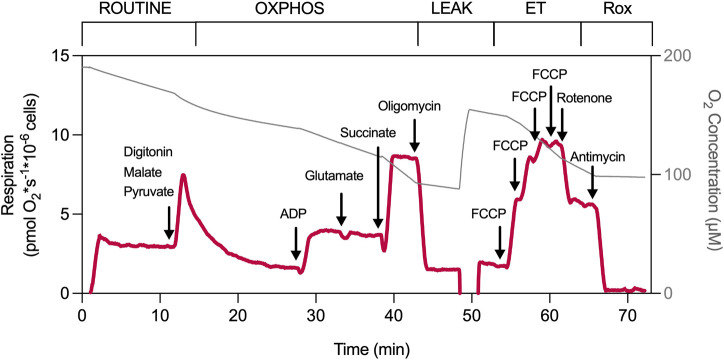
Experimental protocol of permeabilized PBMCs. Mitochondrial respiration was measured by high-resolution respirometry using a substrate–uncoupler–inhibitor titration (SUIT) protocol (Timon-Gomez, 2025; Doerrier et al., 2018; Sjovall et al., 2013a; Gnaiger, 2009). After establishing ROUTINE respiration (intact cells), PBMCs were permeabilized with digitonin (at a predetermined optimized dose, usually between 3–6 µg/million cells) in the presence of CI substrates malate and pyruvate (5 mM, respectively). Oxidative phosphorylation (OXPHOS) was stimulated by the addition of ADP (1 mM), followed by glutamate (5 mM). The addition of the CII substrate succinate (10 mM) enabled convergent electron transfer via both CI and CII. OXPHOS was inhibited by the ATP synthase inhibitor oligomycin (1 μg/mL), revealing LEAK respiration. Maximal decoupled respiration of the electron transfer system (ET capacity) was induced by stepwise titration (0.5 µM steps) of the protonophore FCCP. The inhibition of CI by rotenone (2 µM) revealed the decoupled respiration via CII. The residual oxygen consumption (Rox) was induced by the addition of the CIII inhibitor antimycin A (1 μg/mL). Induced respiratory states and respiratory substrates, uncoupler, and inhibitors used are indicated in and above the graph.

As sample amount is often a limitation, the comprehensive SUIT protocol in permeabilized PBMCs described here was initially established in platelets to acquire as much information as possible about the respiratory capacity from a single experiment (Figure 4) (Sjovall et al., 2013a; Gnaiger, 2009; Sjovall et al., 2013b).

Following stabilization of ROUTINE respiration (intact cells), digitonin (at a predetermined optimized dose, see below) is added to permeabilize the plasma membrane together with the complex I substrates malate and pyruvate. After approximately 15 min, ADP is added to trigger phosphorylation, followed by the addition of another complex I substrate glutamate (N_*P*_) to bypass possible limitations in pyruvate metabolism. Subsequent succinate addition clearly increases phosphorylating respiration by convergent electron transfer via complex I and complex II (NS_*P*_). The LEAK state (NS_*L*_) is induced by the ATP synthase inhibitor oligomycin (1 μg/mL). Maximal decoupled respiratory capacity (NS_*E*_) is reached by titration of the protonophore FCCP. With optimal digitonin permeabilization of the plasma membrane, the *P*/*E* ratio is approximately 1.0, indicating no flux limitation by the phosphorylating system in PBMCs at saturating exogenous complex I and II substrates. The maximal decoupled complex II-related capacity (S_*E*_) is visible after inhibition of complex I by rotenone, followed by Rox induced by complex III inhibition by antimycin. Selected respiratory parameters of PBMCs freshly isolated from whole blood from a large control cohort are provided in Table 3. They comprise updated and reanalyzed data derived from our previously published control cohorts (Westerlund et al., 2024).

**TABLE 3:** Respiratory rates and ratios of digitonin-permeabilized PBMCs isolated from freshly drawn whole blood from healthy volunteers.

Selected respiratory parameter	All participants		Adult participant (age ≥18 years)		Child participant (age <18 years)	
	Cell count normalized^a^		Cell count normalized^a^		Cell count normalized^a^	
	Median (IQR)	n	Median (IQR)	n	Median (IQR)	n
ROUTINE respiration	3.32 (0.95)	60	3.37 (0.95)	50	3.19 (0.88)	10
Background respiration	1.51 (0.69)	60	1.53 (0.73)	50	1.40 (0.54)	10
N_*P*_ (PM)	7.21 (2.47)	60	7.26 (2.47)	50	7.06 (2.41)	10
N_*P*_ (PMG)	7.02 (2.42)	60	6.98 (2.45)	50	7.37 (2.53)	10
NS_*P*_ (PMGS)	11.66 (3.41)	60	11.83 (3.48)	50	11.47 (3.75)	10
LEAK	1.67 (0.53)	60	1.65 (0.54)	50	1.71 (0.57)	10
NS_*E*_	10.26 (3.87)	60	10.15 (3.89)	50	10.64 (4.17)	10
S_*E*_	4.98 (1.96)	60	5.01 (1.88)	50	4.14 (1.55)	10
P/L	6.95 (2.15)	60	7.08 (2.28)	50	6.52 (1.73)	10
E/L	6.29 (1.74)	60	6.35 (1.88)	50	5.62 (1.54)	10
1–(L/P)	0.86 (0.05)	60	0.86 (0.05)	50	0.85 (0.04)	10

^a^ Respiration measured in pmol O_2_ / s / 10^6^ PBMCs, except for ratios that are independent of cell count and mitochondrial content; IQR, interquartile range. The data consist of updated and reanalyzed data (see Results) derived from our previously published control cohort (Westerlund et al., 2024). For definitions of respiratory rates, ratios and substrates used, see Table 1.

### Evaluation of chamber cell concentration on mitochondrial respiration in permeabilized cells

**FIGURE 5:**
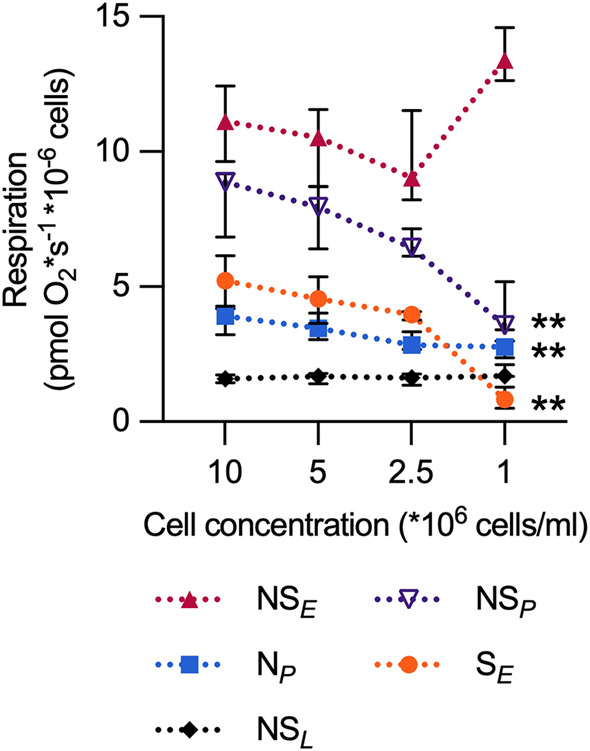
The oxygraph chamber concentration of PBMCs affects several mitochondrial pathway-control states in permeabilized cells. Mitochondrial respiration rates were measured in permeabilized PBMCs using high-resolution respirometry at different pathway-control states induced by a substrate–uncoupler–inhibitor titration (SUIT) protocol at PBMC concentrations ranging from 1 × 10^6^ cells/mL to 10 × 10^6^ cells/mL. At 1 × 10^6^ cells/mL, respiration rates at most pathway-control states were significantly lower than at 10 × 10^6^ cells/mL. n = 6. **p < 0.01 compared to the 10 × 10^6^ PBMCs/mL group. Data were analyzed by the Friedman test followed by Dunn’s multiple comparison test and plotted as median with interquartile range (IQR). For definitions of respiratory rates and ratios, see Table 1.

Mitochondrial respiration per million cells at decreasing PBMC concentrations in the oxygraph chambers was also evaluated in the permeabilized condition. At the lowest cell concentration, 1 × 10^6^ cells/mL, respiration rates at most pathway-control states were significantly lower than at 10 × 10^6^ cells/mL (Figure 5). This supported the corresponding findings in intact cells and reaffirmed the decision to perform experiments at ≥5 × 10^6^ PBMCs/mL in the Oroboros oxygraph.

### The influence of blood storage on respiratory parameters in permeabilized PBMCs

**FIGURE 6:**
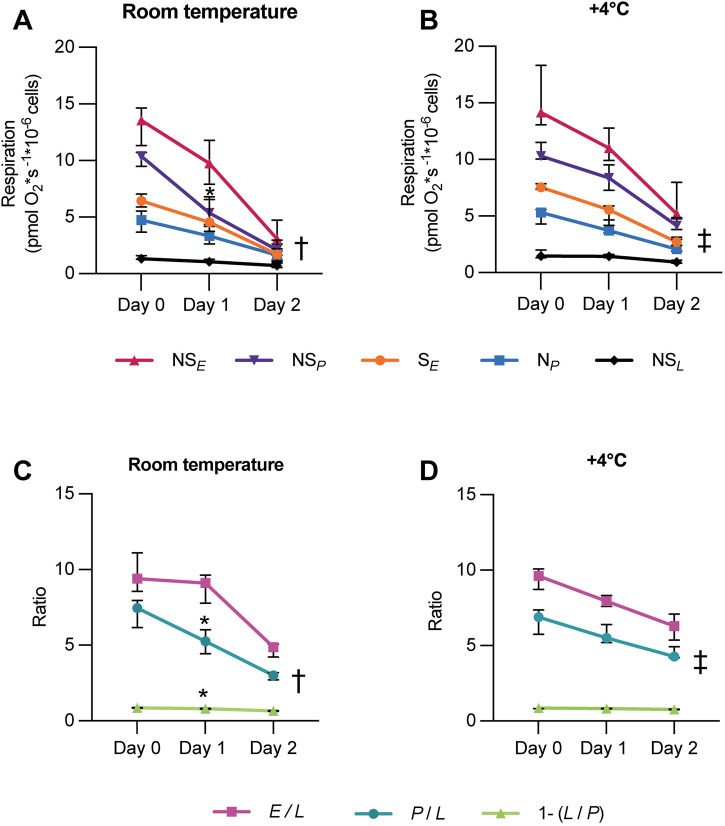
Storage of PBMCs over 2 days decreases mitochondrial respiratory rates at different pathway states in permeabilized cells following density gradient isolation. Leukocyte suspensions were stored at **(A,C)** room temperature or **(B,D)** +4 °C on a tilting board. PBMCs were isolated and analyzed immediately (Day 0), after 1 day (Day 1), and after 2 days (Day 2). Mitochondrial respiratory rates were measured in permeabilized PBMCs using high-resolution respirometry after assessing the optimal digitonin dose. Results indicate that storage decreased both ET and OXPHOS capacities **(A,B)** and selected flux control ratios and efficiencies **(C,D)** within 2 days, and some within 1 day. Data were analyzed by the Friedman test followed by Dunn’s multiple comparison test and plotted as median with interquartile range (IQR). n = 6. For definitions of respiratory parameters, see Table 1. † p < 0.001 for all parameters compared to the Day 0 group. ‡ p < 0.01 or 0.001 compared to the Day 0 group.

Similar to the intact cells (above), the effect of storage on mitochondrial respiration of permeabilized PBMCs was evaluated in cells stored as leukocyte suspensions on a tilting board at room temperature or +4 °C (Figure 6). The electron transfer (ET) capacities of both the succinate-linked pathway (S_*E*_) and the convergent NADH- and succinate-linked pathway (NS_*E*_), as well as the OXPHOS capacities of the NADH-linked pathway (N_*P*_), convergent NADH- and succinate-linked pathway (NS_*P*_), and calculated ratios, were significantly decreased after 2 days of storage (Figures 6A–D). In addition, the LEAK respiration of the convergent NADH- and succinate-linked pathway (NS_*L*_) was also significantly decreased after 2 days. Furthermore, NS_*P*_ and the ratios P/L and 1−(L/P) were already significantly decreased after 1 day of storage at room temperature (Figures 6A,C). Similar alterations in rates and ratios due to delayed isolation were obtained in PBMCs stored and isolated from whole blood resting at room temperature (data not shown).

### Digitonin titration in PBMCs

**FIGURE 7:**
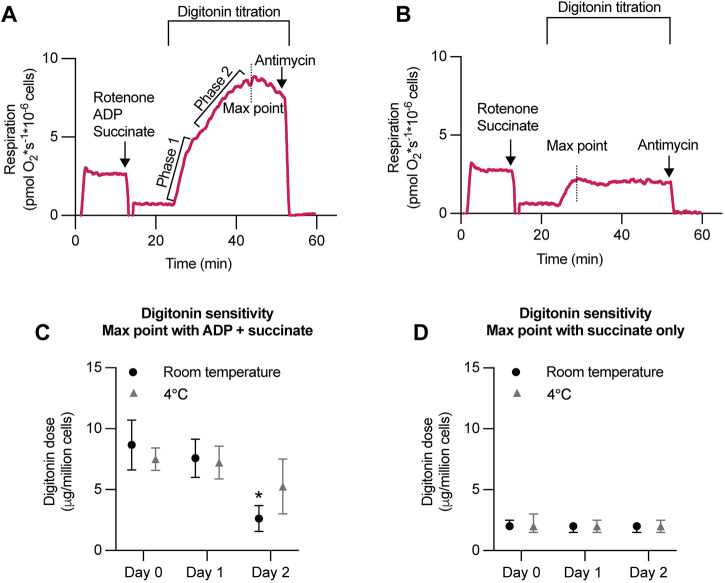
Digitonin titration in PBMCs exhibits a biphasic pattern in the respiration response at saturating concentrations of ADP and succinate and reveals an altered digitonin sensitivity after storage. **(A,B)** Representative traces of digitonin titration in PBMCs, isolated from leukocyte suspensions, at a concentration of 10 × 10^6^/mL suspended in MiR05 buffer. After stabilization at ROUTINE respiration, complex I was inhibited by 2 μM rotenone. Simultaneously, **(A)** 10 mM succinate and 1 mM ADP, or **(B)** 10 mM succinate, were added, followed by stepwise (5 μg) digitonin titration until no further increase in mitochondrial respiration was detected (Max point). The residual oxygen consumption (Rox) was determined by adding the complex III inhibitor antimycin A (1 μg/mL). At saturating concentrations of ADP and succinate **(A)**, digitonin titrations exhibited a biphasic pattern and a Max point sensitive to storage **(C)**. Digitonin titration with succinate only **(B)** showed a maximum permeability (Max point) at a lower digitonin concentration than succinate together with ADP, and a Max point insensitive to 2 days of storage **(D)**. Data were analyzed by the Friedman test followed by Dunn’s multiple comparison test and plotted as median with interquartile range (IQR). n = 7.

Digitonin titration experiments were performed to select the optimal dose of digitonin in mitochondrial respiration measurements using PBMCs (Figure 7). At saturating concentrations of ADP and succinate, digitonin titration resulted in a biphasic pattern of the respiration response, while titrations in the presence of succinate only did not (Figures 7A,B). The digitonin dose at which the respiration rate reached its maximum (Max point) was lower in titrations performed with succinate only than in titrations with succinate together with ADP (Figures 7C,D). In addition, the Max point with both ADP and succinate was significantly lower after storage for more than 2 days at room temperature, while the Max point with only succinate remained at a similar level.

**FIGURE 8:**
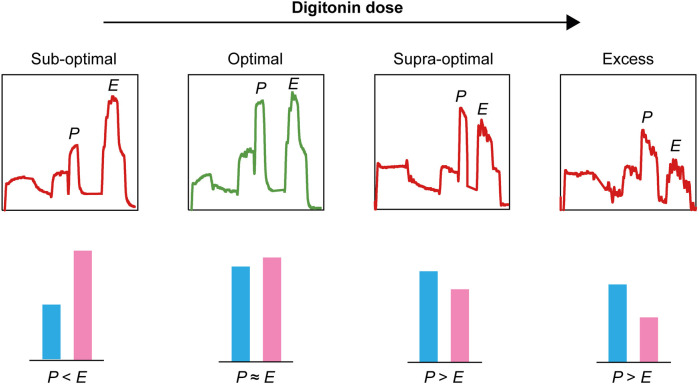
Schematic (based on representative Oroboros traces) of the interrelationship between OXPHOS capacity (*P*) and ET capacity (*E*) at sub-optimal, optimal, supra-optimal, and excess doses of digitonin applied in the presented SUIT protocol in permeabilized PBMCs. At saturating concentrations of substrates and ADP, *P* should be comparable to *E* in PBMCs (Timon-Gomez, 2025). Selecting a sub-optimal dose of digitonin will not adequately permeabilize the plasma membrane and may result in an underestimation of P, while selecting a supra-optimal dose potentially will lead to an underestimation of E. Considering the biphasic pattern of digitonin titration in PBMCs and the sensitivity to storage, a careful assessment of the optimal digitonin dose for each experimental setting is crucial to achieve correct measures of respiratory rates. Note that selecting the digitonin-induced maximal respiration (Max point) reached in PBMCs in the presence of ADP and succinate will result in a supra-optimal or excessive dose.

Furthermore, to illustrate the influence of digitonin dose on mitochondrial respiratory patterns in PBMCs, increasing doses of digitonin were applied in a set of experiments using a SUIT protocol (Figure 8). Doses were selected based on previously performed titrations (range 1–15 µg/million cells). Specifically, the interrelationship between OXPHOS capacity (*P*) and ET capacity (*E*) was depicted and highlighted. As *P* should be comparable to *E* (*E*/*P* ≈ 1.0, see Figure 4 above) at saturating concentrations of substrates and ADP, the dose of digitonin at which this occurred was referred to as the optimal dose. Doses lower than the optimal showed *P* < *E,* and doses higher than the optimal showed *P* > *E.* In addition, at an excess digitonin dose, both *P* and *E* were decreased compared to the optimal dose.

## Discussion

Here, we share methodological aspects of assessing mitochondrial respiratory function in PBMCs using the Oroboros oxygraph. In 2007, with support from Oroboros Instruments, we developed our first comprehensive protocols aimed at extracting as much information as possible from whole blood samples from patients and controls. We selected two protocols, one for intact cells and a substrate–uncoupler–inhibitor titration (SUIT) protocol for permeabilized cells. Normally, extracting cells from 6–12 mL of whole blood would be sufficient to execute one of these protocols in PBMCs and both protocols in platelets (Sumbalova, 2020; Sjovall et al., 2013b). Less blood is clearly required for platelet analysis than for PBMC analysis. For preterm babies, umbilical cord blood, and newborns, we naturally receive very limited blood volumes. On these occasions, the permeabilized protocol in platelets has been prioritized because less blood volume is needed, platelets constitute a more homogeneous cell (cell fragment) population, and mitochondrial function can be evaluated in greater detail without substrate supply being rate-limiting. Initially, patients with sepsis admitted to the intensive care unit were sampled (Sjovall et al., 2010), followed by publication of our methodological experience using platelets (Sjovall et al., 2013b). We have since applied blood cell respirometry to a variety of diseases and conditions. The overall goal is to determine how the bioenergetic capacity of mitochondria within blood cells could be of use to study disease mechanisms, provide diagnostic support of primary mitochondrial disease (PMD), and possibly be used to monitor therapeutic intervention.

In addition to several studies of sepsis (Sjovall et al., 2014; Sjovall et al., 2010; Sjovall et al., 2013a) and a case study on 2,4-dinitrophenol intoxication (Lindeman et al., 2026), we have investigated patients with amyotrophic lateral sclerosis (ALS) (Ehinger et al., 2015), Huntington’s disease (Ehinger et al., 2016), and PMD (Westerlund et al., 2018). Furthermore, we have studied the correlation of mitochondrial respiration in platelets, peripheral blood mononuclear cells, and muscle fibers (Westerlund et al., 2024) and presented an account of mitochondrial function in peripheral blood cells across the human lifespan (Ehinger et al., 2024).

### The SUIT protocol

Many additional oxygraph protocols have been evaluated and developed since 2007 (championed by Erich Gnaiger, Oroboros Instruments) to assess additional and detailed aspects of respiratory capacity, including pathway control, fatty acid oxidation, and sample condition (Timon-Gomez, 2025; Gnaiger, 2020b; Gnaiger, 2020a). A single, straightforward, and simple SUIT protocol has pros and cons. The SUIT protocol presented here does not evaluate fatty acid metabolism, and, as we are mainly using fresh samples, it normally does not include cytochrome *c* to test the integrity of the mitochondrial outer membrane. Another specific limitation is the evaluation of S_*P*_ and N_*E*_, as these, in the presented protocol, are derived only from the relative contribution of complex I and II substrates during convergent electron transfer. With optimal digitonin concentration, the *P*/*E* ratio is approximately 1.0, indicating no limitation by the phosphorylating system in PBMCs at the selected concentrations of complex I and II substrates combined. Therefore, if complex I- and complex II-linked electron transfer display similar and complete additivity, S_*E*_ (only CII-linked electron transfer after rotenone addition) and S_*P*_ (calculated as NS_*P*_-N_*P*_, i.e., CII-linked phosphorylating electron transfer in the presence of CI-linked substrates) assessed in the protocol should be identical. Using our historical data of >300 patients and controls (Westerlund et al., 2024), we find that S_*E*_ is significantly higher than S_*P*_ for PBMCs and platelets, indicating that the additivity is not complete under these experimental conditions (Supplementary Figure S3 and data not shown). This is in agreement with Timon-Gomez (2025), who concluded that incomplete additivity suggests that combined capacities leading into the Q-junction are higher than the transfer capacity downstream of Q. However, we propose that calculating the relative contribution of complex I and II during convergent electron transfer could be of possible value in the diagnostic setting (Westerlund et al., 2018). Depending on sample availability, further diagnostic protocols can be added or used depending on the experimental conditions and research questions at hand. In a recent study using human PBMCs, 20 respiratory states were captured using only two SUIT reference protocols run in parallel (Timon-Gomez, 2025). As our protocol has been maintained unaltered since 2007, we direct the reader to the protocols presented by Timon-Gomez (2025) (see PBMC protocol details in the legend to Figure 2) and the information available on Bioblast MitoPedia, an open-access knowledge database maintained by Oroboros Instruments (https://www.bioblast.at/index.php/PBMC). Of particular importance are updates in substrate and inhibitor concentrations, for example, oligomycin, where the recommendation now is ≤50 nM, malate that should be used at ≤ 2 mM concentration, and rotenone at 0.5 µM (not 0.5 mM, which is a typographical error in Timon-Gomez (2025)). Furthermore, to address the issue of limited sample availability, a smaller chamber (0.5 mL instead of the standard 2 mL) has been developed for the Oroboros oxygraph (Leo et al., 2025), which offers a significant advantage for studies with limited amounts of sample or low respiratory capacities.

#### Digitonin permeabilization and storage of samples

As mentioned above, maximal phosphorylating respiration is similar to maximal decoupled respiration in PBMCs (the *P*/*E* ratio is approximately 1.0), also confirmed by Timon-Gomez (2025). We demonstrate here that selecting a sub-optimal dose of digitonin will not adequately permeabilize the plasma membrane and may result in an underestimation of *P*, while selecting a supra-optimal dose potentially will lead to an underestimation of *E*. The selection of the optimal dose is more difficult in PBMCs than in platelets, as the former display a biphasic pattern during digitonin titration. Selecting the maximal respiration (Max point) reached in PBMCs in the presence of ADP and succinate will result in a supra-optimal or excessive dose. Therefore, following guidance from the classical digitonin titration (Doerrier et al., 2018), the selected dose (in our experience, between 3–6 µg/million cells) should be tested in an actual SUIT protocol assessing both maximal phosphorylating respiration (NS_*P*_) and maximal decoupled respiration (NS_*E*_). Reviewing more than 300 samples in patients and controls, we found that >30% of our SUIT protocols in PBMCs displayed lower (>20%) NS_*E*_ than NS_*P*_. The conclusion is that the dose of digitonin used (the standard was 6 µg/million PBMCs) was too high for a subset of these samples, and we recommend that careful assessment of the optimal digitonin dose for each experimental setting be performed to achieve correct measures of respiratory rates.

Storing samples resting in whole blood or leukocyte suspension at room temperature will clearly affect respiratory capacities, which is in agreement with Macleod (2025), and there were similar findings for storage at +4 °C. Furthermore, the sample sensitivity to permeabilization increased with storage time. In addition, the phenotype of the resultant isolate following storage is clearly aberrant after 48 h, with a decreased proportion of lymphocytes and an increased proportion of non-lymphoid cells of the isolated PBMC population. Importantly, alteration in the phenotypic composition of the PBMC population or platelet contamination (Sumbalova, 2020; Rausser et al., 2021) could influence the assessed mitochondrial capacities (Rausser et al., 2021). Based on our findings, we suggest isolating and assessing mitochondrial capacities within 24 h, which is in agreement with Johnson et al. (2022). One alternative is to isolate and cryopreserve PBMCs for future use, as demonstrated by Korandova (2025).

#### Respiratory capacities in PBMCs from healthy controls

Here, as guidance for future research, we provide updated and reanalyzed data derived from our previously published control cohorts (Westerlund et al., 2024) (all assessed using the presented protocols). Experiments with sub-optimal (P/E < 0.8) permeabilization and supra-optimal doses of digitonin (E/P < 0.8) were removed, and additional parameters were extracted, including separated data on our pediatric control cohort for the SUIT protocol (Tables 2, 3).

#### Blood cell respiration as a “liquid biopsy”

The bioenergetics of the PBMC population has emerged as a surrogate marker of mitochondrial function in diseases not classically defined by immune dysregulation, such as type 2 diabetes mellitus, obesity, cardiovascular diseases, fatigue, and depression, and in neurodegenerative disorders including Alzheimer’s and Parkinson’s diseases (Mahapatra, 2025; Varada, 2025; Herpich, 2025; Gumpp, 2025; Zhou, 2025; Carvalho, 2025; de Melo, 2025). In these contexts, the mitochondrial OXPHOS capacity of PBMCs has been championed as a minimally invasive window into bioenergetic dysfunction occurring in anatomically more inaccessible tissues, such as the myocardium or central nervous system. However, this approach has limitations, as we have found that even the within-tissue correlation of mitochondrial respiration between PBMCs and platelets is weak. Furthermore, neither PBMCs nor platelet respiration correlated significantly with muscle tissue respiration (Westerlund et al., 2024). In agreement with this, in non-disease conditions, Martin Picard’s group has shown that neither mitochondrial function nor mitochondrial DNA density correlated between tissues in animals, which points to independent, tissue-specific energy-sensing pathways (Devine et al., 2025). Therefore, it is becoming clear that respirometry of blood cells should not be used as a universal biomarker for less accessible tissues or as a signature for systemic mitochondrial function in healthy controls (Rausser et al., 2021; Westerlund et al., 2024). However, all diseases and conditions affecting mitochondria in blood could potentially be diagnosed or monitored using blood cell respirometry. Examples are primary mitochondrial disease (with sufficient mutation load in blood cells), drug intoxications, poisonings, and evaluation of therapeutic intervention (Westerlund et al., 2024; Sjovall et al., 2014; Sjovall et al., 2010; Sjovall et al., 2013a; Westerlund et al., 2018; Bungatavula et al., 2025; Jang et al., 2018; Weiss et al., 2022; Lindeman et al., 2026).

We conclude that OXPHOS capacity in PBMCs can reproducibly be assessed *ex vivo* using comprehensive protocols, but that great care must be taken during permeabilization to achieve correct measures of respiratory rates.

## Data Availability

The raw data supporting the conclusions of this article will be made available by the authors, without undue reservation.
