# Supplementary material for: Mitochondrial respiration in human peripheral blood mononuclear cells: methodology and influence of permeabilization and storage
Source: Front Mol Biosci. 2026 Mar 13;13:1756657. doi: 10.3389/fmolb.2026.1756657 (PMC13021407; doi:10.3389/fmolb.2026.1756657)
Supplement: Supplementary file 1 [file DataSheet1.pdf]

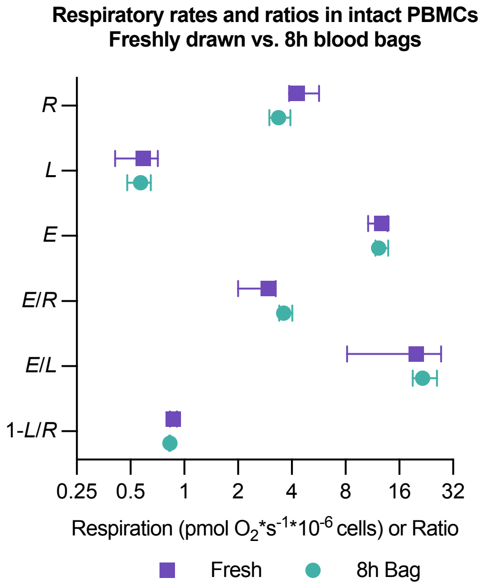

S1. Mitochondrial respiratory rates and ratios in intact PBMCs isolated from either leukocyte suspensions obtained using the Reveos 3C System® (8h Bag) or isolated from freshly drawn whole blood (Fresh). Mitochondrial respiratory rates were measured in intact cells using high-resolution respirometry at three different respiratory coupling states induced in a coupling control protocol. For the definition of respiratory rates, see Table 1. Results indicate that mitochondrial respiration in intact PBMCs isolated from leukocyte suspensions obtained using the Reveos 3C System® within 8h from blood donation displayed similar respiratory rates and ratios as compared to intact PBMCs freshly isolated from whole blood. Median with interquartile range (IQR). Freshly drawn blood: n=26, 8h leukocyte suspension: n=10.

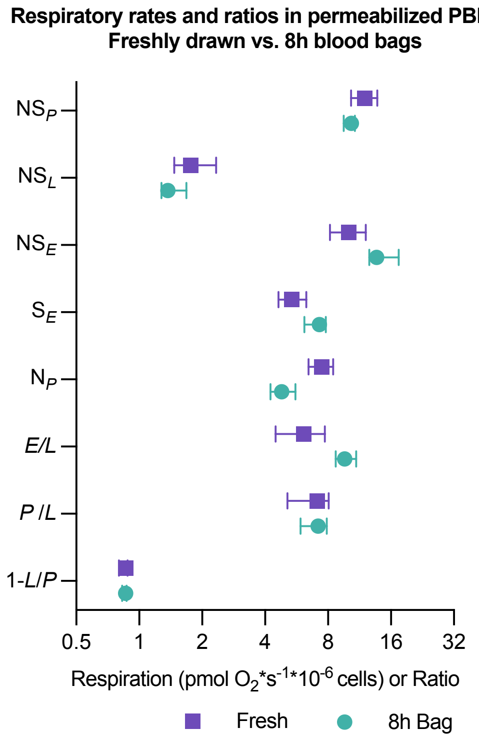

S2. Mitochondrial respiratory rates and ratios in permeabilized PBMCs isolated from either leukocyte suspensions obtained using the Reveos 3C System® (8h Bag) or isolated from freshly drawn whole blood (Fresh). Mitochondrial respiratory rates were measured in digitonin-permeabilized cells using high-resolution respirometry at three different respiratory coupling states induced in a coupling control protocol. For the definition of respiratory rates, see Table 1. Results indicate that mitochondrial respiration in permeabilized PBMCs isolated from leukocyte suspensions obtained using the Reveos 3C System® within 8h from blood donation displayed similar respiratory rates and ratios as compared to permeabilized PBMCs freshly isolated from whole blood. Median with interquartile range (IQR). Freshly drawn blood: n=26, 8h leukocyte suspension: n=10.

PBMC permeabilized protocol - comparison of CII flux rates

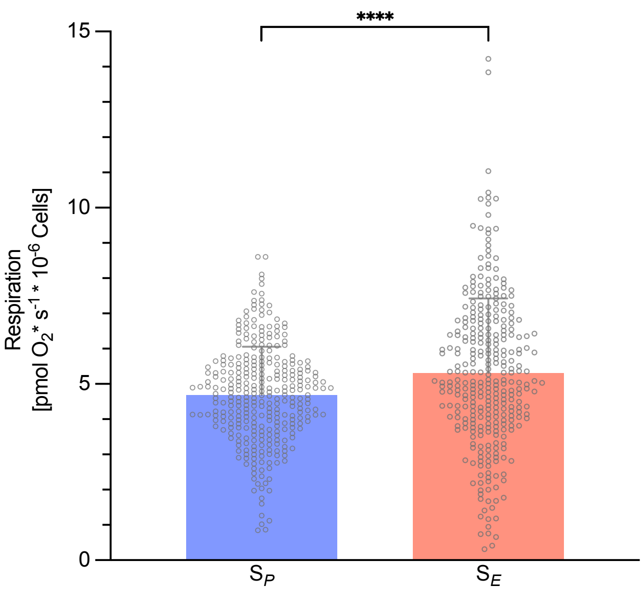

S3. In the presented SUIT protocol PBMCs display a P/E-ratio of ≈ 1.0. Therefore, if complex I- and complex II-linked respiratory capacities display similar and complete additivity in both the coupled and non-coupled state, S_E (only CII-linked electron transport) and S_P (CII-linked electron transport in the presence of CI-linked substrates during convergent electron transfer) should be identical. Here, using our historical data of 317 patients and controls [1], we find that S_E is 13.3% higher than S_P indicating that the additivity is not complete under these experimental conditions. ****$p<0.0001$ paired Student's *t* test.

1. Westerlund, E., et al., *Correlation of mitochondrial respiration in platelets, peripheral blood mononuclear cells and muscle fibers.* Heliyon, 2024. **10**(5): p. e26745.
